# Polymyalgia Rheumatica Complicated by Nephrotic Syndrome in a Nonagenarian: A Case Report

**DOI:** 10.7759/cureus.66592

**Published:** 2024-08-10

**Authors:** Ayato Nakagawa, Natsumi Yamamoto, Chiaki Sano, Ryuichi Ohta

**Affiliations:** 1 Family Medicine, International University of Health and Welfare, Tokyo, JPN; 2 Community Care, Unnan City Hospital, Unnan, JPN; 3 Community Medicine Management, Shimane University Faculty of Medicine, Izumo, JPN

**Keywords:** rural, general medicine, family medicine, systemic inflammation, renal dysfunction, elderly, prednisolone, nephrotic syndrome, polymyalgia rheumatica

## Abstract

This case report describes a 91-year-old bedridden man with a complex medical history who presented with fever and low oxygen saturation, suspected to be aspiration pneumonia. Further investigation revealed nephrotic syndrome, microscopic hematuria, and joint pain. The diagnosis of polymyalgia rheumatica (PMR) was considered due to the presence of characteristic symptoms and elevated inflammatory markers despite the inability to perform a kidney biopsy. The patient was treated with low-dose prednisolone (PSL), leading to significant improvement in joint pain, renal function, and overall condition. This case highlights the importance of considering PMR in elderly patients with unexplained nephrotic syndrome and systemic inflammation. Early diagnosis and corticosteroid treatment can improve clinical outcomes and enhance activities of daily living. This report underscores the need for awareness of PMR as a potential cause of nephrotic syndrome in the elderly and the effectiveness of PSL in managing such cases.

## Introduction

Polymyalgia rheumatica (PMR) is a disease characterized by moderate-to-severe musculoskeletal pain and stiffness in the neck, shoulder, and hip joints [1]. The etiology is unknown and typically affects individuals over 50, with incidence increasing with age [2]. PMR is often associated with giant cell arteritis (GCA), with 40%-60% of GCA patients having PMR and 16%-21% of PMR patients developing GCA [3]. Few cases report PMR patients with renal dysfunction due to amyloid A (AA) amyloidosis or diffuse proliferative glomerulonephritis [4].

Diagnosing PMR lacks a gold standard, relying on the European College of Rheumatology/American College of Rheumatology (EULAR/ACR 2012) classification criteria, including age over 50, new shoulder pain, and elevated erythrocyte sedimentation rate or C-reactive protein. Oral steroids, particularly prednisolone (PSL; 12.5-25 mg daily), are the primary treatment [5]. This case study discusses a 91-year-old bedridden man with fever, low oxygen saturation, nephrotic syndrome, microscopic hematuria, and arthralgia, leading to a PMR diagnosis complicated by nephrotic syndrome. Despite communication challenges preventing a kidney biopsy, he was treated with PSL. This case report explores diagnosing and managing nephrotic syndrome as a PMR complication.

## Case presentation

A 91-year-old bedridden man from a nursing home presented to a rural community hospital with complaints of fever and low oxygen saturation, which had started the previous night. Suspecting aspiration pneumonia, his family doctor transferred the patient immediately to the hospital. His medical history included diabetes, posterior ligament ossification, lumbar spinal canal stenosis, hypertension, chronic kidney disease (CKD), bilateral lower limb vein thrombosis, constipation, and neurogenic bladder. His medications included pregabalin of 75 mg daily, lubiprostone of 24 mg daily, edoxaban of 30 mg daily, linagliptin of 5 mg daily, olmesartan of 10 mg daily, and amlodipine of 5 mg daily.

Upon admission, his vital signs were temperature 37.1°C, blood pressure 137/72 mmHg, pulse 76 times/minute, SpO_2_ 88% (room air), and respiratory rate 12 breaths/minute. Physical examination revealed generalized edema, inspiratory rales, and decreased alveolar breath sounds in the right lung field. A joint examination showed tenderness on bilateral shoulders, elbows, wrists, knees, and lateral thighs. Laboratory tests indicated leukocytosis, renal impairment, hypermagnesemia, hypoalbuminemia, elevated immunoglobulin (Ig) M and IgA, anemia, low ferritin levels with the urinalysis of positive proteinuria, hematuria, and glucosuria without casts (Table 1).

**Table 1:** Initial laboratory data of the patient IU: international unit; eGFR: estimated glomerular filtration rate; C3: complement 3; C4: complement 4; HPF: high-power field

Parameter	Level	Reference range
White blood cell count （10^3^/μL）	10.60	3.5-9.8
Red blood cell count （10^6^/μL）	2.50	4.10-5.30
Hemoglobin （g/dL）	7.5	36-48
Platelet count （10^4^/μL）	24.4	13.0-36.9
Aspartate aminotransferase （IU/L）	17	8-38
Alanine aminotransferase （IU/L）	8	4-44
Alkaline phosphatase （U/L）	125	38-113
Total protein （g/dL）	7.7	6.6-8.1
Albumin （g/dL）	2.2	3.9-4.9
Glucose (mg/dL)	117	70-110
Urea nitrogen (mg/dL)	6.4	3.0-6.9
Blood urea nitrogen	37.4	8.0-20.0
Creatinine (mg/dL)	2.32	0.40-1.10
eGFR (mL/minute/1.73m^2^)	21.1	~60.0
Serum Na (meq/L)	140	135-147
Serum K (meq/L)	4.3	3.3-4.8
Serum Cl (meq/L)	105	98-108
Serum Ca (mg/dL)	7.7	8.8-10.2
Serum P (mg/dL)	3.1	2.7-4.6
Serum Fe （mg/dL)	24	54-181
Ferritin (ng/mL)	91.3	31-325
Immunoglobulin G （mg/dL)	2,734	870-1,700
Immunoglobulin A （mg/dL)	590	110-410
Immunoglobulin M （mg/dL)	152	35-220
Vitamin B12 (pg/mL)	973	187-883
Folic acid (ng/mL)	6.1	3.1-20.5
C3 （mg/dL）	112	86-160
C4 （mg/dL）	22	17-45
Urine testing	-	-
Leukocyte	3+	Negative
Protein	3+	Negative
Nitrite	Negative	Negative
Glucose	1+	Negative
Urobilinogen	Negative	Negative
Occult blood	+	Negative
Bilirubin	Negative	Negative
Ketone	Negative	Negative
Specific gravity	1.011	-
White blood cells	10-19/HPF	-
Red blood cells	＞50/HPF	-
Microbe	1+	-
Urine protein (actual measurement)	420	Negative

**Figure 1:**
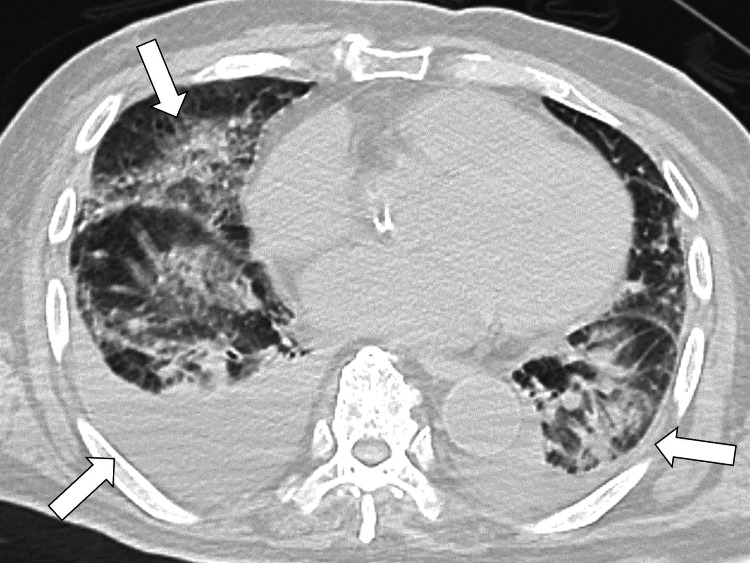
CT of the chest showing infiltrative shadows and pleural effusions in bilateral lungs (white arrows) CT: computed tomography

Chest X-ray and computed tomography (CT) revealed infiltrative shadows in the right lung and bilateral pleural effusion (Figure 1).

**Figure 2:**
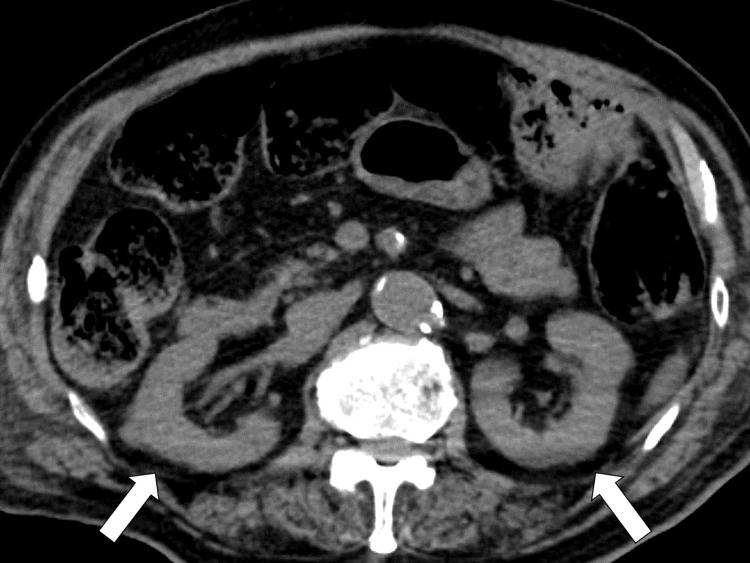
CT of the abdomen showing bilateral renal atrophy (white arrows) CT: computed tomography

Abdominal CT showed bilateral renal atrophy (Figure 2).

Gram staining of urine and sputum samples showed gram-positive cocci and gram-negative bacilli. The clinical diagnosis was bacterial pneumonia, and the patient was started on ceftriaxone (1 g/day). Additionally, considering the findings of microscopic hematuria, nephrotic syndrome, CKD, transudative pleural effusion, and chronic heart failure, furosemide (40 mg/day) was administered. A kidney biopsy was deemed unsafe due to the patient’s inability to remain still.

For anemia, sodium ferrous citrate was initiated, but two units of red blood cells were transfused due to progression. Regarding joint pain, joint ultrasound showed synovial thickening and increased blood flow in the wrists. The wrist X-ray showed no joint space narrowing or erosion. Rheumatoid factor, anticitrullinated protein antibodies, antineutrophil cytoplasmic antibodies, and antinuclear antibodies were negative, as were tests for hepatitis B and syphilis. Bence-Jones protein was negative. Elevated urinary N-acetyl-β-D-glucosaminidase and β2 microglobulin indicated renal tubular damage (Table 2).

**Table 2:** Added laboratory data of the patient TSH: thyroid stimulating hormone; T3: triiodothyronine; T4: tetraiodothyronine; HBs: hepatitis B surface; HCV: hepatitis C virus; HIV: human immunodeficiency virus; CH: measuring the 50% hemolytic complement; PR3: proteinase3; MPO: myeloperoxidase; S/CO: sample/cutoff; ANCA: antineutrophil cytoplasmic antibody

Parameter	Level	Reference range
TSH （μU/mL）	2.46	0.35-4.94
Free T3 （pg/mL）	1.7	1.88-3.18
Free T4 （ng/dL）	1.1	0.7-1.48
HBs antigen （IU/mL）	0.00	0.00-0.04
HCV antibody （S/CO）	0.14	0-0.99
Treponema pallidum antibody (U/mL)	0.0	~10.0
Rapid plasma regain test (unit)	0.0	~1.0
HIV antigen/antibody （S/CO）	0.20	~0.99
Rheumatoid factor (U/mL)	2	~15
Anticyclic citrullinated peptide antibody (U/mL)	<0.6	<5
Antinuclear antibody	＜40	＜40
CH50 （CH50/mL）	34.4	25.0-48.0
PR3-ANCA （U/mL）	<1.0	<1.0
MPO-ANCA （U/mL）	<1.0	<1.0
Urine testing		
Protein （mg/dL）	336	-
Protein （g/day）	5.71	-
Glucose (g/day)	1.07	-
Glucose (mg/dL)	63	-
Immunoglobulin G （mg/dL）	78	-
N-acetyl-β-D-glucosaminidase （IU/L）	21.2	0.7-11.2
β_2_ microglobulin (μg/L)	27,379	~289

The patient was clinically suspected of PMR based on the 2012 EULAR/ACR provisional classification criteria for PMR [2]. Complicated with nephrotic syndrome, PSL (10 mg/day) was started. Within three days, the patient’s joint symptoms significantly improved. Renal function and urinary protein levels also gradually improved. After one month of treatment, the patient's overall condition had sufficiently improved, and he was discharged to the previous healthcare facility.

## Discussion

This case report presents a case of PMR complicated by nephrotic syndrome. The absence of bacterial or viral infections typically associated with nephrotic syndrome suggested a link to PMR. Although a kidney biopsy was not feasible due to the patient's condition, the diagnosis was supported by meeting PMR criteria. This case indicates that low-dose PSL can effectively alleviate PMR symptoms and improve renal function in patients with PMR-associated nephrotic syndrome.

Reports of PMR complicated by nephrotic syndrome are rare, and it remains unclear whether PMR precedes nephrotic syndrome or vice versa. In our case, the older patient had PMR with suspected nephrotic syndromes not investigated in depth. Takeshima et al. reported a case of diffuse intracapillary proliferative glomerulonephritis associated with PMR and nephrotic syndrome, while Javaid et al. described type AA amyloidosis with nephrotic syndrome following PMR onset [6,7]. These cases suggest that systemic inflammation from PMR can potentially trigger nephrotic syndrome.

The proposed mechanisms include activated immune cells and kidney amyloid deposition due to chronic inflammation. Prior studies have shown immune complexes in PMR patients' synovium, perimysium, and temporal arteries [8,9]. Secondary AA amyloidosis, a reaction in chronic inflammatory diseases, commonly deposits AA amyloid in the kidneys [10,11]. In our case, the patient’s inflammatory conditions might not be investigated for long because of frailty and ageism. Such a condition might cause a nephrotic syndrome in the patient. Continual immune activation and AA amyloid deposition likely contribute to PMR patients' glomerular inflammation and renal damage, so general physicians should be careful about the presence of chronic inflammation in older patients and investigate them effectively.

Treatment of PMR with nephrotic syndrome may require prolonged PSL therapy, so general physicians should be meticulous in the diagnosis of PMR. Like PMR, nephrotic syndrome needs steroid therapy, and Japanese guidelines define complete remission of the nephrotic syndrome as urinary protein <0.3 g/day, with treatment effectiveness assessed at one and six months [12,13]. While this patient's PMR symptoms and renal dysfunction improved with low-dose PSL, proteinuria persisted, indicating the need for continued steroid treatment. In cases of PMR complicated with nephrotic syndrome, they may need prolonged PSL for remission, so general physicians should manage their symptoms and complications from steroids comprehensively for a better quality of life.

Elderly patients discharged with new or additional impairments in activities of daily living (ADLs) after acute illness face higher mortality and permanent disability risks. Factors like hypoalbuminemia, polypharmacy, malnutrition, fall risk, and delirium contribute to decreased ADLs during hospitalization [14-16]. In this case, intensive investigations such as a kidney biopsy are impossible due to the patient's communication challenges and restlessness in rural contexts [17,18]. However, excluding autoimmune and infectious causes through blood tests and physical examinations can lead to the diagnosis of PMR with nephrotic syndrome. Early intervention can also improve the patient's condition, joint pain, and ADLs. Prompt diagnosis and treatment in general medicine are crucial for stabilizing the patient's condition, preventing delirium, facilitating early mobilization, and enhancing ADLs [19,20].

## Conclusions

This case report highlights a rare instance of PMR complicated by nephrotic syndrome in a 91-year-old bedridden patient. Despite the inability to perform a kidney biopsy, the diagnosis was made based on clinical criteria and the exclusion of other causes. Treatment with low-dose PSL significantly improved both PMR symptoms and renal function. This case emphasizes the importance of considering PMR in elderly patients with unexplained nephrotic syndrome and systemic inflammation. Early diagnosis and corticosteroid intervention can substantially improve clinical outcomes and ADLs.
